# Seroprevalence and risk factors of herpes simplex virus type-2 infection among pregnant women in Northeast India

**DOI:** 10.1186/1471-2334-11-325

**Published:** 2011-11-23

**Authors:** Dipankar Biswas, Biswajyoti Borkakoty, Jagadish Mahanta, Kamini Walia, Lahari Saikia, Brogen S Akoijam, Lobsang Jampa, Alia Kharkongar, Eric Zomawia

**Affiliations:** 1 Regional Medical Research Centre, North East Region (Indian Council of Medical Research), Post Box-105, Dibrugarh-786001, Assam, India; 2 Indian Council of Medical Research, Department of Health Research, (Ministry of Health & Family Welfare), Ansari Nagar, New Delhi-110029, India; 3 Assam Medical College & Hospital, Dibrugarh-786001, Assam, India; 4 Regional Institute of Medical Sciences, Lamphel-795004, Manipur, India; 5 Directorate of Health Services, Naharlagun, Govt. of Arunachal Pradesh, India; 6 Ganesh Das Hospital, Shillong-793001, Meghalaya, India; 7 Aizawl Civil Hospital, Aizawl, Mizoram, India

## Abstract

**Background:**

Herpes simplex virus type-2 (HSV-2) is one of the most common sexually transmitted infections that facilitate human immunodeficiency virus (HIV) acquisition by over two fold or more. The development of HSV-2 control methods as a measure to control HIV epidemic in high HSV-2/HIV areas has become a priority. Two out of the six high HIV prevalent states of India are located in the Northeastern region of India. Due to lack of documented HSV-2 studies from this part of the country; there was a need for estimating the seroprevalence and risk factors of HSV-2 infection in this defined population.

**Methods:**

Pregnant women (n = 1640) aged18 years and above attending antenatal clinics of tertiary referral hospitals in five Northeastern states of India were screened for type specific HSV-2 IgG antibodies. Blood samples were collected from all the participants after conducting interviews. Univariate and multivariate analyses were performed to identify the risk factors associated with HSV-2 seropositivity.

**Results:**

Overall seroprevalence of HSV-2 infection was 8.7% (142/1640; 95% CI 7.3-10.0) with a highest prevalence of 15.0% (46/307; 95% CI 11.0-19.0) in the state of Arunachal Pradesh. Higher seroprevalence was observed with increasing age (Adj. Odds Ratio [AOR] 1.9 for 22-25 years old, AOR 2.29 for > 29 years old). The risk factors associated with HSV-2 seropositives were multiple sex partners (AOR 2.5, *p *= 0.04), condom non-user's (AOR 4.7, p *<*0.001), early coitarchal age (age of first intercourse) 'less than 18 years' (AOR 9.6, *p = *0.04), middle income group (AOR 2.1, *p = *0.001) compared to low income group and low level of education (AOR 3.7, *p = *0.02) compared to higher education. HSV-2 seropositivity was higher among Christians (12.6%) compared to Muslims (3.8%). The most frequent clinical symptoms among HSV-2 seropositives were excess vaginal discharge in last one year (53.5%, 76/142) and pelvic pain (26.1%, 37/142). While among subjects with genital ulcers, HSV-2 seroprevalence was 36.8% (7/19).

**Conclusions:**

Overall seroprevalence of HSV-2 infection among pregnant women of Northeast India is relatively low. The generation of awareness among high risk groups may have played key role to limit the infection. The role of vaccination against HSV-2 in near future and elimination of HSV-2 viral shedding along with genital tract inflammation in high HIV/HSV-2 areas may be an option for initiating successful intervention strategies to reduce the transmission and acquisition of HIV infection in Northeast India.

## Background

Herpes Simplex Virus Type-2 (HSV-2) infections is almost always sexually transmitted and is the common cause of genital ulcer disease (GUD) worldwide [1,2]. It is also a good marker of sexual behavior in the population [1,2]. Seroprevalence studies show wide variations in infection rates by geographic location. Highest prevalence of HSV-2 has been found in some parts of Africa, America and the lowest in Asia [3]. Globally about 535.5 million were infected with HSV-2 with an overall prevalence of 16.2% in 2003 [4]. The HSV-2 prevalence in South-Asia in 2003 was estimated to be 29.4% and 33.2% among males and females in the age group of 15-49 years respectively [4]. Recent study from India shows HSV-2 prevalence among general population to be around 10% [5]. HSV-2 is gaining special attention as a significant risk factor for acquisition of human immunodeficiency virus type-1 (HIV-1). HSV-2 infection is also present in 30 to 70% of those in Europe and 50 to 90% of those in Africa among patients with HIV infection [6]. HSV-2 increases the risk of HIV-1 transmission by more than two fold and HIV transmission on a per-sexual act basis by up to fivefold and may account for 40 - 60% of new HIV infections in high HSV-2 prevalent populations [7-14]. It is assumed that infection with HSV-2 disrupts the genital mucosa and provides a portal of entry for HIV, leading to increased susceptibility of HIV in HIV-negative persons. Also, HSV-2 is thought to enhance HIV acquisition due to dense inflammatory infiltrates of CD4/CD8/dendritic cells in the genital tract associated with HSV-2 shedding [15]. Moreover, in HIV-positive persons, infection with HSV-2 accelerates replication and genital shedding of the virus, thus such individuals are more likely to transmit HIV [16,17]. Increasing evidence of HSV-2 facilitating HIV acquisition and transmission makes the development of HSV control methods a priority [1,6]. Also, other meta-analysis data from general population among women showed that in prevalent HSV-2 infection, HIV acquisition increased by over a three-fold [18]. A study by Nagot *et al*. highlights the association of HSV-2 with significantly higher viral load of HIV-1 in plasma and in genital secretions in women with sexually acquired HIV-1 [17]. This finding has direct clinical implications, suggesting that elimination of HSV-2 viral shedding and genital tract inflammation can reduce the transmission and acquisition of HIV infection.

The role of HSV-2 as a biological cofactor in HIV acquisition and transmission, which may have contributed substantially to HIV infections particularly by facilitating HIV spread among the low-risk population with stable long-term sexual partnerships [19]. A study conducted in four cities of Africa indicated that in the two West African cities with lower HSV-2 prevalence, HIV remained concentrated in higher risk groups. However, in the cities with high HSV-2 prevalence, HIV transmission was observed in a large fraction of the population [20]. These results suggest that HSV-2 may be the trigger for the explosive expansion of HIV and were also corroborated by the epidemiological synergy study of HIV and HSV-2 [19]. The increasing evidence that HSV-2 facilitates HIV acquisition and transmission and the synergistic effect of HSV-2 on HIV, makes the development of HSV-2 control methods a priority.

In Northeast India, the epidemic of HIV-1 is driven mainly by the IDU population in the State of Manipur, Mizoram and Nagaland. The state of Manipur has the highest HIV prevalence in the country (1.67% in 2006) [21]. In a recent study from this Institute, the HIV-1 prevalence was found to vary between 23%-32% among the IDUs of Manipur [22]. Female sex workers (FSWs) are common in scattered locations in few northeast states of India. One recent study from this Institute has found HIV-1 prevalence of 13.6% in FSWs of Nagaland, a state in northeast India (unpublished data of the authors). The population of Northeast India is heterogeneous and there is a huge socio-cultural difference among the states. HIV preventive measures have been the cornerstone in the efforts to halt the epidemic amongst IDUs and FSWs of Northeast India. Over the years, HIV has made in-roads into the general population of the Northeastern states. The average HIV prevalence among women attending antenatal clinics in India is 0.48%. The HIV prevalence in the antenatal clinic attendees in the Northeastern state of Manipur was 1.25% and in Mizoram was 1.0% in 2006, which was among the highest in the country in this group of population [21].

Considering the above facts, this study was conducted to assess the seroprevalence of HSV-2 and its risk factors among pregnant women (antenatal clinic attendees) in five North-eastern states of India (viz. Assam, Arunachal Pradesh, Manipur, Meghalaya, and Mizoram). Since, more than 75% of primary genital herpes virus infections are asymptomatic [23], the seroepidemiological studies are critical in understanding the pattern and distribution of infection within the population to understand the HSV-2 related HIV transmission dynamic in the different communities of Northeast India.

## Methods

### Study area and Population

This is a cross-sectional study which was conducted in five, out of eight Northeastern states of India to understand the epidemiology and role of risk factors associated with HSV-2 seroprevalence with an aim focused towards prevention. A total of 1640 pregnant woman attending antenatal clinics (ANCs) in five tertiary referral hospitals representing the general population of each of the five Northeastern states i.e., Arunachal Pradesh, Assam, Manipur, Meghalaya, and Mizoram were enrolled in the study between April 2007 and March 2009. The present study was approved by institutional human ethical committee of the Regional Medical Research Centre. Trained field investigators had enrolled pregnant women aged 18 years and above who had consented for the study. Pregnant women in labor or under serious medical conditions were excluded from the study.

### Data collection

A predesigned and pretested structured questionnaire having information on socio-demographic variables such as age, religion, education, occupation, type of family, geographic location, annual income, gestational weeks, and risk factors that may be associated with sexually transmitted diseases like number of sex partners in previous one year, use of condom, alcohol consumption, coitarchal age, commercial sex worker, and type of sex partner were recorded. The clinical history, signs & symptoms associated with genito-pelvic infections, including previous history of genital ulcer, pelvic pain, and excessive vaginal discharge, pain during sexual intercourse, genital warts, and swelling in the groin were also recorded. The participants were interviewed confidentially and examined by qualified gynaecologists.

### Specimen collection and laboratory test

Specimen of 5 ml venous blood were collected from all the enrolled subjects in K3 EDTA tubes and immediately kept at +4°C. Plasma samples were separated on the same day and stored at -20°C freezer in aliquots until analyzed. Plasma samples were subjected for HSV-2 IgG detection using type specific HSV-2 glycoprotein (gpG2) IgG EIA kit (HerpeSelect 2 ELISA IgG, Focus Diagnostics, USA) as per manufacture's manual. The cut-off value used to determine a positive test on this kit was > 1.10. Index value between 1.1 and 0.9 were considered equivocal and index values below 0.9 were considered negative as per the kit instruction manual. The equivocal samples were re-tested with the same kit. The samples that were found to be equivocal in the repeat test, a second samples were collected from the same subjects after 1-2 months for reconfirmation. All the positive samples and randomly selected 10% negative samples were re-tested for confirmation.

### Statistical analyses

Data were entered and analyzed in SPSS version 13.0 statistical software (SPSS, Chicago, USA). The statistical significance of HSV-2 seroprevalence with other associated factors (socio-demographic, risk variables and clinical findings) was obtained using univariate analysis. The multivariate analysis was performed by binary logistic regression using Wald Backward elimination method with probability for stepwise entry at *p *= 0.05 and removal at *p *= 0.1, to find the association between categorical variables with HSV-2 seroprevalence. Odds ratios (OR) were calculated using Binary logistic regression analysis where first category (having lowest seroprevalence) was used as a reference in each group. OR and their 95% confidence intervals (95% CI) were documented to indicate magnitude and direction of associations. The variables showing significant interactions were analyzed separately. The level of statistical significance was set at *p *≤ 0.05.

## Results

A total of 1640 pregnant women from five Northeastern states of India were studied. The median age of the study subjects was 25 years (SD ± 4.7) and ranged from 18 to 46 years. The literacy rate of the enrolled subjects was 84.5% (n = 1386), among which 49.3% (n = 809) were educated up to primary level. Majority of the subjects were housewife 79.9% (n = 1310), lived in nuclear family 57.9% (n = 950), and were from low income group 71.8% (n = 1177). Within the enrolled subjects, 67.0% (n = 1099) were in 3^rd ^trimester of pregnancy and 43.2% (n = 708) were primi-gravida with a median of two gravida (2 ± 1.8).

State-wise, socio-demographic variables of the study subjects are listed in Table 1. The demographic composition in the present study reveals that Christians accounted for the major chunk of subjects in the state of Mizoram (96.1%), Meghalaya (70.0%) followed by Arunachal Pradesh (35.2%), whereas Hindus followed by Muslims were found to be the major religion of the study cohort in Assam (90.3% and 8.7%) and Manipur (86.1% and 13.9%) respectively. Also, it was observed that 84.9% of the enrolled Muslim subjects were from low income group compared to 49.4% and 39.5% in Hindus and Christians respectively. Overall 11.7% (192/1640) enrolled subjects were migrants with highest number in Arunachal Pradesh (19.9%) followed by Meghalaya (19.5%) and Mizoram (10.8%).

**Table 1:** State wise socio-demographic variables of the study subjects, Northeast India (n = 1640).

Variables (total)	Manipur n (%)	Assam n (%)	Meghalaya n (%)	Arunachal Pradesh n (%)	Mizoram n (%)
**Subjects recruited (1640)**	402	300	400	307	231
**Age groups**					
18 to 21 yrs (355)	75(18.7)	77(25.7)	145(36.2)	30(9.8)	28(12.1)
22 to 25 yrs (592)	125(31.1)	137(45.6)	113(28.3)	140(45.6)	77(33.3)
26 to 29 yrs (385)	103(25.6)	63(21.0)	77(19.2)	85(27.7)	57(24.7)
> 29 yrs (308)	99(24.6)	23(7.7)	65(16.3)	52(16.9)	69(29.9)
**Religion**					
Hindu (844)	346(86.1)	271(90.3)	93(23.2)	125(40.7)	9(3.9)
Christian (611)	--	1(0.3)	280(70.0)	108(35.2)	222(96.1)
Muslim (106)	56(13.9)	26(8.7)	7(1.8)	17(5.5)	--
Others (79)	--	2(0.7)	20(5.0)	57(18.6)	--
**Occupation**					
House wife (1310)	341(84.8)	293(97.7)	328(82.0)	168(54.7)	180(77.9)
Regular employee (110)	7(1.7)	1(0.3)	17(4.2)	79(25.7)	6(2.6)
Unskilled worker (75)	39(9.7)	6(2.0)	50(12.5)	37(12.1)	43(18.6)
Business (45)	15(3.7)	--	5(1.3)	23(7.5)	2(0.9)
**Education**					
Primary & less (1063)	252(62.7)	231(77.0)	278(69.5)	166(54.1)	136(58.9)
Secondary (462)	105(26.1)	66(22.0)	101(25.2)	98(31.9)	92(39.8)
Graduate & above (115)	45(11.2)	3(1.0)	21(5.3)	43(14.0)	3(1.3)
**Type of family**					
Nuclear (950)	232(57.7)	144(48.0)	238(59.5)	211(68.7)	125(54.1)
Joint (616)	169(42.0)	147(49.0)	156(39.0)	42(13.7)	102(44.2)
Single (74)	1(0.3)	9(3.0)	6(1.5)	54(17.6)	4(1.7)
**Income group**					
Low (1177)	247(61.4)	262(87.3)	324(81.0)	160(52.1)	184(79.6)
Middle & high (463)	155(38.6)	38(12.67)	76(19.0)	147(47.88)	47(20.35)
**Migrant labor **(192)	14(3.5%)	14(4.7%)	78(19.5)	61(19.9%)	25(10.8%)
**Duration of Pregnancy**					
1-12 weeks (28)	--	9(3.0)	19(4.8)	--	--
13-28 weeks (513)	100(24.9)	205(68.3)	131(32.7)	39(12.7)	38(16.5)
29-42 weeks (1099)	302(75.1)	86(28.7)	250(62.5)	268(87.3)	193(83.5)
**Commercial sex worker **(8)	2(0.5%)	--	--	5(1.6%)	1(0.4%)

### Seroprevalence

The overall HSV-2 seroprevalence was 8.7% (142/1640; 95% CI 7.3-10.0) with a highest prevalence of 15.0% (95% CI 11.0-19.0) in the state Arunachal Pradesh followed by Mizoram at 13.4% (95% CI 9.0-17.8), Meghalaya at 10.5% (95% CI 7.5-13.5), Assam at 4.0% (95% CI 1.8-6.2) and Manipur at 2.7% (95% CI 1.1-4.3). The increasing HSV-2 seroprevalence was observed with the increasing age e.g. 6.5% seroprevalence in '18-21 years' age group to 9.4% in '29 years and above' with a significant peak of 10.5% in '22-25 years' (Table 2). HSV-2 seroprevalence was 17.9% (approaching significance *p *= 0.06) in 1st trimester of pregnancy compared to 6.8% in 2nd and 9.3% in 3rd trimester. Though HSV-2 seroprevalence was higher among migrants (12.0%) compared to the locals (8.2%), it was not significant statistically (*p *= 0.08).

**Table 2:** Risk factors associated with HSV-2 seroprevalence among pregnant women in Northeast India (n = 1640).

Characteristics (n)	HSV-2 Sero Prevalence n (%)	Univariate analysis			Multivariate analysis		
		OR	95% **C.I**.	*p *value	AOR	95% **C.I**.	*p *value
**Age groups**							
18 to 21 yrs (355)	23 (6.5)	Ref	--	--	Ref	--	--
22 to 25 yrs (592)	62 (10.5)	1.69	1.03-2.78	0.04	1.90	1.09-3.31	0.02
26 to 29 yrs (385)	28 (7.3)	1.13	0.64-2.01	0.67	1.52	0.81-2.87	0.19
> 29 yrs (308)	29 (9.4)	1.50	0.85-2.65	0.16	2.29	1.19-4.39	0.01
**States**							
Manipur (402)	11 (2.7)	Ref	--	--	Ref	--	--
Assam (300)	12 (4.0)	1.48	0.64-3.40	0.36	2.30	0.96-5.48	0.06
Meghalaya (400)	42 (10.5)	4.17	2.11-8.22	< 0.001	5.13	2.52-10.43	< 0.001
Mizoram (231)	31 (13.4)	5.51	2.71-11.2	< 0.001	4.51	2.15-9.49	< 0.001
Arunachal Pradesh (307)	46 (15.0)	6.27	3.19-12.3	< 0.001	11.49	5.18-25.48	< 0.001
**Occupation**							
Unskilled worker (75)	12 (6.9)	Ref	--	--	Ref	--	--
House wife (1310)	105 (8.0)	1.18	0.64-2.20	0.59	1.55	0.80-3.00	0.20
Business (45)	6 (13.3)	2.09	0.74-5.92	0.17	2.28	0.72-7.20	0.16
Regular employee (110)	19 (17.3)	2.84	1.32-6.11	0.008	3.45	1.35-8.81	0.01
**Education**							
Graduate & above (115)	6 (5.2)	Ref	--	--	Ref	--	--
Secondary (462)	42 (9.1)	1.82	0.75-4.38	0.18	2.57	0.92-7.19	0.07
Primary & less (1063)	94 (8.8)	1.76	0.75-4.12	0.19	3.73	1.29-10.75	0.02
**Type of family**							
Joint (616)	35 (5.7)	Ref	--	--	Ref	--	--
Nuclear (990)	99 (10.4)	1.93	1.30-2.88	0.001	1.66	1.08-2.57	0.02
Single (74)	8 (10.8)	2.01	0.90-4.52	0.090	1.72	0.64-4.64	0.28
**Income group**							
Low (1177)	85 (7.2)	Ref	--	--	Ref	--	--
Middle (463)	57 (12.3)	1.80	1.27-2.57	0.001	2.15	1.39-3.31	0.001
**Coitarchal age**							
> 30 yrs (29)	1 (3.4)	Ref	--	--	Ref	--	--
18 to 30 yrs (1335)	91 (6.8)	2.05	0.28-15.2	0.48	2.36	0.29-19.40	0.43
< 18 yrs (218)	48 (22.0)	7.91	1.05-59.6	0.045	9.59	1.13-81.65	0.04
No response (58)	2 (3.4)	1.00	0.09-11.5	1.00	0.60	0.05-7.83	0.70
**Alcohol use**							
Never user (1267)	97 (7.7)	Ref	--	--	Ref	--	--
Regular & occasional users (373)	45 (12.1)	1.66	1.14-2.41	0.008	1.18	0.74-1.86	0.49
**Sex partner**							
Single (1601)	134 (8.4)	Ref	--	--	Ref	--	--
More than 1 (39)	8 (20.5)	2.83	1.27-6.27	0.01	2.50	1.02-6.13	0.045
**Condom use**							
Regular & occasional users (341)	18 (5.3)	Ref	--	--	Ref	--	--
Never user (1299)	124 (9.5)	1.9	1.14-3.15	0.01	4.73	2.51-8.91	< 0.001

**AOR: **Adjusted Odds Ratio; **95% CI: **95% confidence interval; **Coitarchal age: **age at first intercourse

### Risk Factors

The univariate and multivariate analysis of risk factors associated with HSV-2 seroprevalence is presented in Table 2. In univariate analysis, it was found that HSV-2 seroprevalence differs significantly (*p *< 0.001) among the subjects of different religion in North-east India. Subjects from Christian community were at higher risk (AOR 3.5, 95% CI 1.2-10.1, *p *= 0.02) of getting infection with reference to Muslims (analyzed separately because of significant interaction with 'state'). On multivariate analysis, it was found that subjects in the state of Arunachal Pradesh has the highest risk of HSV-2 compared to the state of Manipur (AOR 11.5, 95% CI 5.2-25.5, *p *< 0.001). The risk-factors that had a significant association with HSV-2 were condom never users compared to regular or occasional users (AOR, 4.7, 95% CI 2.5-8.9, *p *< 0.001); coitarchal age less than 18 years (AOR 9.6, 95% CI 1.1-81.6, *p *= 0.04); more than one sex partner (AOR 2.5, 95% CI 1.1-6.1, *p *= 0.04); middle or high income compared to low income subjects (AOR 2.2, 95% CI 1.4-3.3, *p = *0.001); subjects from nuclear family (AOR 1.7, 95% CI 1.1-2.6, *p *= 0.02); subjects with low level of education (AOR 3.7, 95% CI 1.3-10.7, *p *= 0.02) and subjects having regular earning through a regular job (AOR 3.4, 95% CI 1.3-8.8, *p *= 0.01) [see table 2]. Though HSV-2 seroprevalence was higher amongst alcoholics (12.1%) compared to non-alcoholics (7.7%), it was not significant in multivariate analysis.

The most frequent clinical symptoms among HSV-2 seropositives were excess vaginal discharge in last one year (53.5%, 76/142) and pelvic pain (26.1%, 37/142). HSV-2 seroprevalence was highest, 36.8% (7/19; 95% CI 15.2-58.5) among subjects with genital ulcers. The association of clinical signs & symptoms with HSV-2 seroprevalence is presented in Table 3. Significantly higher HSV-2 seroprevalence was observed among the subjects with genital ulcer (AOR 4.1, 95% CI 1.4-12.2, *p *= 0.01), history of pelvic pain (AOR 2.2, 95% CI 1.4-3.5, *p *= 0.001) and cervicitis (AOR 2.1, 95% CI 1.4-3.4, *p *= 0.001).

**Table 3:** Association of clinical sign & symptoms with HSV-2 seroprevalence among pregnant women of Northeast India (n = 1640).

Clinical sign & symptoms	Response/findings (n)	HSV-2 Sero prevalence n (%)	Univariate analysis			Multivariate analysis		
			OR	95% CI	*P* value	AOR	95% CI	*P* value
**Cervicitis**	No (1447)	107 (7.4)	Ref	--	--	Ref	--	--
	Yes (193)	35 (18.1)	2.80	1.8-4.2	< 0.001	2.14	1.36-3.38	0.001
**Genital ulcer**	No (1621)	135 (8.3)	Ref	--	--	Ref	--	--
	Yes (19)	07 (36.8)	6.42	2.5-16.6	< 0.001	4.11	1.38-12.21	0.011
**Pelvic pain**	No (1423)	105 (7.4)	Ref	--	--	Ref	--	--
	Yes (217)	37 (17.1)	2.58	1.72-3.87	< 0.001	2.20	1.40-3.45	0.001
**Excess vaginal discharge**	No (1003) Yes (637)	66 (6.6) 76 (11.9)	Ref 1.92	-- 1.36-2.72	-- < 0.001	Ref 1.46	-- 1.00-2.15	-- 0.053

**AOR: **Adjusted Odds Ratio; **95% CI: **95% confidence interval

## Discussion

The conceptual importance of this study was largely based on the increasing evidence that HSV-2 infection has a synergistic activity on acquiring HIV infection and this has been well documented [6-14,16,17]. Since there were virtually no data on the prevalence of HSV-2 in the community of Northeastern states of India, there was a need to know the prevalence of this infection to plan an intervention strategy especially in the high HIV prevalent states of Northeast India. This sero-epidemiological study was based on detection of HSV-2 IgG antibodies against type specific glycoprotein G2. The HerpeSelect HSV-2 IgG ELISA test (Focus Diagnostics), in an evaluation study demonstrated a sensitivity of 96% in a group of pregnant women and 95% in a STD population of men and women. The specificity of Focus HSV-2 ELISA was also high in these groups i.e. 97% in pregnant women and 96% in the STD population [24]. The relative specificity of the assay as compared to Western Blot assay (Gold standard) in a low prevalent population was shown to be 98.7% and relative sensitivity 100% as per instruction manual of the kit. Though, the kit used in our study has a very high sensitivity and specificity, the false positivity of HSV-2 in low prevalence area cannot be ruled out. We also acknowledge the limitations of statistical test used in this cross-sectional study which may be prone to residual confounding effect in comparison to case control studies.

Our study detected a significant geographical variation of HSV-2 seroprevalence which has also been observed in an earlier study [22]. Community based studies conducted elsewhere in India also reported wide variations in seroprevalence [3,25]. One study from Chennai based on study of around 30 communities, reported a wide variation in prevalence of HSV-2 from 4.1 to 49.1% with a mean prevalence of 15.3% [3]. Studies conducted among the high risk groups showed highest seroprevalence (40% to 70%) [12,26-28]. In our study, we expected highest HSV-2 prevalence in the state of Manipur, which has the highest HIV prevalence in India and expected lowest in the state of Arunachal Pradesh which has the lowest HIV prevalence in the country [21]. But to the contrary, we detected the lowest HSV-2 seroprevalence in the state of Manipur (2.7%) and highest in the state of Arunachal Pradesh (15%). This may be the result of the widespread public awareness in the state of Manipur in last two decades on practice of safe sex to fight HIV epidemic in that state, whereas such public awareness drive is virtually absent in the state of Arunachal Pradesh.

In comparison to other National and international studies, our finding of 8.7% HSV-2 seroprevalence was relatively low. A community based study in New Delhi reported 8.6% seroprevalence among females, which is similar to our findings [25]. Higher seroprevalence were reported from Chennai (15.3%) in South India [3], central India (12.4%) [29] and Gujarat (23.3%) in Western India among women attending gynaecology OPD [30]. Seroprevalence of 7.5% among pregnant women from Jammu & Kashmir, North India [31], and 7.0% among females from Andhra Pradesh, South India was reported in a community based study [32]. The National Health and Nutrition Examination Survey (NHANES) surveillance data from USA found HSV-2 seroprevalence of 20.9% in US females aged '14 to 49 years', with an overall seroprevalence of 16.2% [33]. The HSV-2 seroprevalence among 15 to 49 years females in South Asian region was reported to be 33.2%, whereas it was 19.4% globally [4].

In comparison to low income group, we observed significantly higher HSV-2 seroprevalence among middle or high income group. Studies in India and abroad also observed association of socio-economic status (SES) with HSV-2 seroprevalence [3,25,34]. This has been observed from both the previously mentioned study from New Delhi [25], and Chennai [3]. In the study from New Delhi, HSV-2 seroprevalence was found to be more common in the urban middle class community with higher average monthly income (12.6%) as compared to urban slum with lower average monthly income (3.1%) [25]. While, the study from Chennai reported an independent association of community-level factors like SES with HSV-2 sero-prevalence [3]. In another study from Australia, found that HSV-2 seroprevalence was significantly lower in areas of low SES than in high SES areas among both men and women for all ages [34]. However, some other studies have reported significant association between HSV-2 seropositivity and lower income [35]. Though this paradox would be hard to explain, it may be related to difference in risk behavior among the different income groups. It was seen in our study that majority of Muslim subjects (84.9%, 90/106) were from low income group. It was also observed that Muslim subjects had the lowest HSV-2 seroprevalence (3.8%) compared to Hindu (5.8%) and Christians (12.6%), which may explain this finding in our study.

It is generally being observed that HSV-2 seroprevalence increases with increasing age [25,33,36,37]. In our study we also observed an increase in prevalence with age (6.5% to 9.4%) but with a peak in prevalence (10.5%) in the 22-25 years age group. This may reflect a relatively recent introduction of HSV-2 in the community which may have resulted in higher prevalence among sexually active younger subjects rather than the older subjects which didn't had the exposure to the virus when they were sexually active in the past.

The risk factors for HSV-2 are well known and include polygamous relationships/multiple sex partners', unsafe sex, increasing numbers of lifetime partners and association with other STIs or GUD etc [7,16,35,38]. The strongest predictor for infection is a person's number of lifetime sex partners [36]. In our study, it was also observed that subjects with multiple sex partners had significantly higher HSV-2 seroprevalence compared to single sex partner. Moreover, it was seen that higher coitarchal age was associated with a lower chance of HSV-2 infection. The association of coitarchal age less than 18 years or early age of first sex with seroprevalence was also observed in other studies [39,40]. In this study, higher seroprevalence was found among Christians versus Muslims. This difference in prevalence with religion (3.8% in Muslim compared to 12.6% among Christians), may be due to practice of male circumcision at infancy or early childhood by the spouses of the pregnant women among Muslims. Male circumcision lowers the prevalence of HSV-2 or HPV has been reported in some studies [41]. Subjects from nuclear family, middle income group, low level of education, having regular source of income, had a higher risk of HSV-2 infections. Modifiable risk factors included, low coitarchal age, never or low condom usage, and multiple sex partners, which can be incorporated in awareness programs to lower the transmission of HSV-2 in the community.

Our study found a significantly higher HSV-2 seropositive amongst subjects with sign and symptoms of genito-pelvic infections such as genital ulcer, pelvic pain and cervicitis. In our study it was found that HSV-2 prevalence was higher among subjects with excessive vaginal discharge but was not significant statistically. The subjects with history of genital ulcers had the highest HSV-2 seroprevalence among the study variables. Also, it is known that majority of HSV2 seropositive people shed virus whether or not they are aware of recurrent genital lesions. Genital tract inflammation is evident in "asymptomatic shedders" [42]. Studies conducted in Surat and Delhi also observed higher HSV-2 seroprevalence in subjects with vaginal discharge and lower abdominal or low backache [30,43].

In Northeast India, the impact of HSV-2 infection in driving the HIV-1 epidemics is still not clear. Although HSV-2 vaccines are still in clinical trials, acyclovir when given continuously or episodically can reduce recurrence of ulcers in individuals with HSV-2 infection. Though clinical trials in Africa on use of acyclovir did not reduce the risk of linked transmission of HIV-1 to couples, however it showed a reduction of plasma HIV-1 RNA level as well as 73% reduction in occurrence of genital ulcers due to HSV-2 [44]. Multiple studies have shown that a persistent increase of 0.5 log_10 _copy per millilitre in the plasma HIV-1 level is associated with a clinically shortened time for progression to AIDS [6]. The demonstration that daily anti-HSV-2 therapy can reduce the viral load by this amount is thus of direct importance for treatment. The study by Nagot *et al*. underlines the findings of a 1989 study showing that zidovudine plus acyclovir (ACV) was associated with prolonged survival, as compared with zidovudine alone [6]. The data from another controlled trial with HSV-2 suppressive therapy with acyclovir showed no evidence of decreasing the incidence of HIV in Tanzania [45]. This landmark study fails to demonstrate the role of acyclovir in decreasing the transmission of HIV among HSV-2 seropositives. This is in contrary to the fact that acyclovir is activated into a human herpes virus (HHV) DNA polymerase inhibitor exclusively by HHV kinases. It suppresses HIV-1 in HHV coinfected human tissues but not in HHV free tissue or cell cultures [46]. Acyclovir treatment in patients coinfected with HSV/HIV has been observed to alter the disease course and decrease HIV viral load which attributed to indirect effects of HSV suppression on HIV replication [47]. Also, if we accept the fact that HSV-2 increases the risk of HIV acquisition by 2-3 folds, than suppression of HSV-2 by ACV which results in decrease genial ulceration and inflammation would be expected to influence HIV acquisition. Use of antivirals for suppression of HSV-2 for lowering the risk of HIV transmission cannot be overlooked until more light is shed on this topic. Therapeutic options that provide a complete eradication of HSV-2 including suppression of genital inflammation may be more relevant for decreasing HIV transmission in high HSV-2 prevalent regions.

## Conclusions

The findings of this study which is first of its kind in North-east India, has provided a baseline data on HSV-2 seroprevalence and risk factors for future in-depth studies. The data provides a useful epidemiologic signal for a possible impending spike in HIV incidence among the young age group (22-25 years) as is indicated by a higher HSV-2 prevalence in this age group. Targeted public health awareness campaigns directed at high risk groups having multiple sexual partners, condom non-users, early coitarchal age, Christian community etc. are needed to be advocated for initiating successful intervention strategies to control HSV-2 and indirectly limiting HIV epidemic in Northeast India.

## Competing interests

The authors declare that they have no competing interests.

## Authors' contributions

This study was conceived by KW with major contributions to design, drafting and critical revision of the manuscript by DB and JM. DB also performed the statistical analysis. BB performed the laboratory analysis, helped in data analysis and drafting the manuscript. LS, BA, LJ, AK and EZ contributed in collecting data, specimens, state level supervision and helped in drafting the manuscript. All authors approved the final version of the manuscript.

## Pre-publication history

The pre-publication history for this paper can be accessed here:

http://www.biomedcentral.com/1471-2334/11/325/prepub
